# Anti-phospholipid IgG antibodies detected by line immunoassay differentiate patients with anti-phospholipid syndrome and other autoimmune diseases

**DOI:** 10.1007/s13317-018-0106-0

**Published:** 2018-05-29

**Authors:** Cecilia Nalli, Valentina Somma, Laura Andreoli, Thomas Büttner, Peter Schierack, Michael Mahler, Dirk Roggenbuck, Angela Tincani

**Affiliations:** 1 University of Brescia, Brescia, Italy; 2 Research and Development Department, Medipan GmbH, Dahlewitz, Berlin, Germany; 3 Research and Development Department, GA Generic Assays GmbH, Dahlewitz, Berlin, Germany; 4 Institute of Biotechnology, Faculty Environment and Natural Sciences, Brandenburg University of Technology Cottbus-Senftenberg, Universitätsplatz 1, 01968 Senftenberg, Germany; 5 Inova Diagnostics, San Diego, CA USA

**Keywords:** Anti-phospholipid syndrome, Beta2 glycoprotein I, Anti-phospholipid antibody, Domain 1, Phosphatidylglycerol

## Abstract

**Purpose:**

Anti-phospholipid antibodies (aPL) analyzed by line immunoassay (LIA) can recognize beta_2_-glycoprotein I (β_2_GPI) domain 1 (D1) epitopes depending on β_2_GPI binding to distinct phospholipids. The aPL LIA was compared with consensus ELISA to investigate whether both techniques can discriminate anti-phospholipid syndrome (APS) patients from aPL-positive, systemic autoimmune rheumatic diseases (SARD) patients without clinical symptoms of APS and controls.

**Methods:**

Thirty-four APS patients (14 arterial/venous thrombosis, 16 pregnancy morbidity, and 4 both), 41 patients with SARD lacking clinical APS criteria but demonstrating positivity for anti-β_2_GPI (aβ_2_GPI) IgG, and 20 healthy subjects (HS) were tested for aPL to cardiolipin (aCL), phosphatidic acid, phosphatidylcholine, phosphatidylethanolamine, phosphatidylglycerol (aPG), phosphatidylinositol, phosphatidylserine, β_2_GPI, prothrombin, and annexin V by LIA. Samples were also tested for aCL, aβ_2_GPI, aβ_2_GPI-domain 1 (aD1), and aβ_2_GPI-domains 4–5 (aD4–5) by ELISA and for lupus anti-coagulant.

**Results:**

Comparison of LIA with ELISA revealed a good agreement for the consensus criteria aPL aβ_2_GPI and aCL IgG (kappa = 0.69, 0.68, respectively) and a moderate agreement for IgM (kappa = 0.52, 0.49, respectively). Regarding ELISA, aD1/aD4–5 demonstrated the best performance of differentiating APS from asymptomatic SARD [area under the curve (AUC): 0.76]. aPG IgG had the best performance by LIA (AUC: 0.72) not significantly different from aD1/aD4–5. There was a good agreement for aPG IgG with aD1/aD4–5 (kappa = 0.71).

**Conclusions:**

aD1/aD4–5 (ELISA) and aPG IgG (LIA) differentiate APS from SARD patients. PG appears to interact with β_2_GPI of APS patients and exposes D1 thereof for disease-specific aPL binding in LIA.

## Introduction

Anti-phospholipid syndrome (APS) is an autoimmune disorder, clinically characterized by arterial and/or venous thrombosis as well as pregnancy-related complications [1, 2]. The APS can be primary or secondary, depending on the absence or presence of any other related systemic autoimmune rheumatic disease (SARD) such as, e.g., systemic lupus erythematosus (SLE). The APS could be associated with a high risk of death in the rare catastrophic anti-phospholipid syndrome, a rapid and simultaneous multi-organ failure due to generalized thrombosis [3]. Apart from one clinical criterion (vascular thrombosis and/or adverse obstetric event), the revised classification criteria require the persistent detection of anti-phospholipid antibodies (aPL) such as anti-beta_2_ glycoprotein I (aβ_2_GPI), anti-cardiolipin (aCL), and/or autoantibodies interfering with coagulation [lupus anti-coagulant (LAC)] for the diagnosis of APS.

The recommended method to detect aβ_2_GPI and aCL is the enzyme-linked immunosorbent assay (ELISA) using a polystyrene solid phase for autoantigen immobilization. However, aPL testing by ELISA still represents a challenge because of the difficulties in the inter- and intra-assay reproducibility [4, 5]. Furthermore, aβ_2_GPI antibodies detected by ELISA have been reported in healthy adults and children. These data support the hypothesis that “innocent”, non-disease associated aPL could exist, too [6, 7]. The subgroup of pathogenic aβ_2_GPI antibodies seems to be mainly directed versus domain 1 (D1) and not to domains 4 and 5 (D4–5), and their pathogenicity appears to be dependent on their Fc glycosylation [8–12]. Indeed, the former are involved in thrombotic events characteristic of APS, whereas the latter do not interfere with the coagulation process, neither are they associated with other clinical APS manifestations [13].

New assay techniques based on chemiluminescence (CIA) or fluorescence enzyme immunoassays for the detection of APS-specific aPL have emerged [4, 14]. Especially, the CIA system has been shown to reduce the inter-laboratory variability [15].

Of note, a novel line immunoassay (LIA) offering the opportunity to test for several aPL has been reported [16, 21]. This LIA appeared to detect preferably aPL to D1 (aD1) of the patient’s β_2_GPI bound to distinct negatively charged phospholipids. Furthermore, aPL not related to aβ2GPI were detected, too [16]. Altogether, the former and these “non-criteria” IgG and IgM aPL to phosphatidylserine (aPS), phosphatidylinositol (aPI), phosphatidylcholine (aPC), phosphatidylethanolamine (aPE), phosphatidic acid (aPA), phosphatidylglycerol (aPG), annexin V (aAnV), and prothrombin (aPT) could be used for aPL profiling and might be helpful in the clinical differentiation of APS patients [17–20]. Nevertheless, the clinical meaning of “non-criteria” antibodies is still debated and β_2_GPI is generally accepted as the major autoantigenic target recognized by APS-specific aPL [2].

**Fig. 1:**
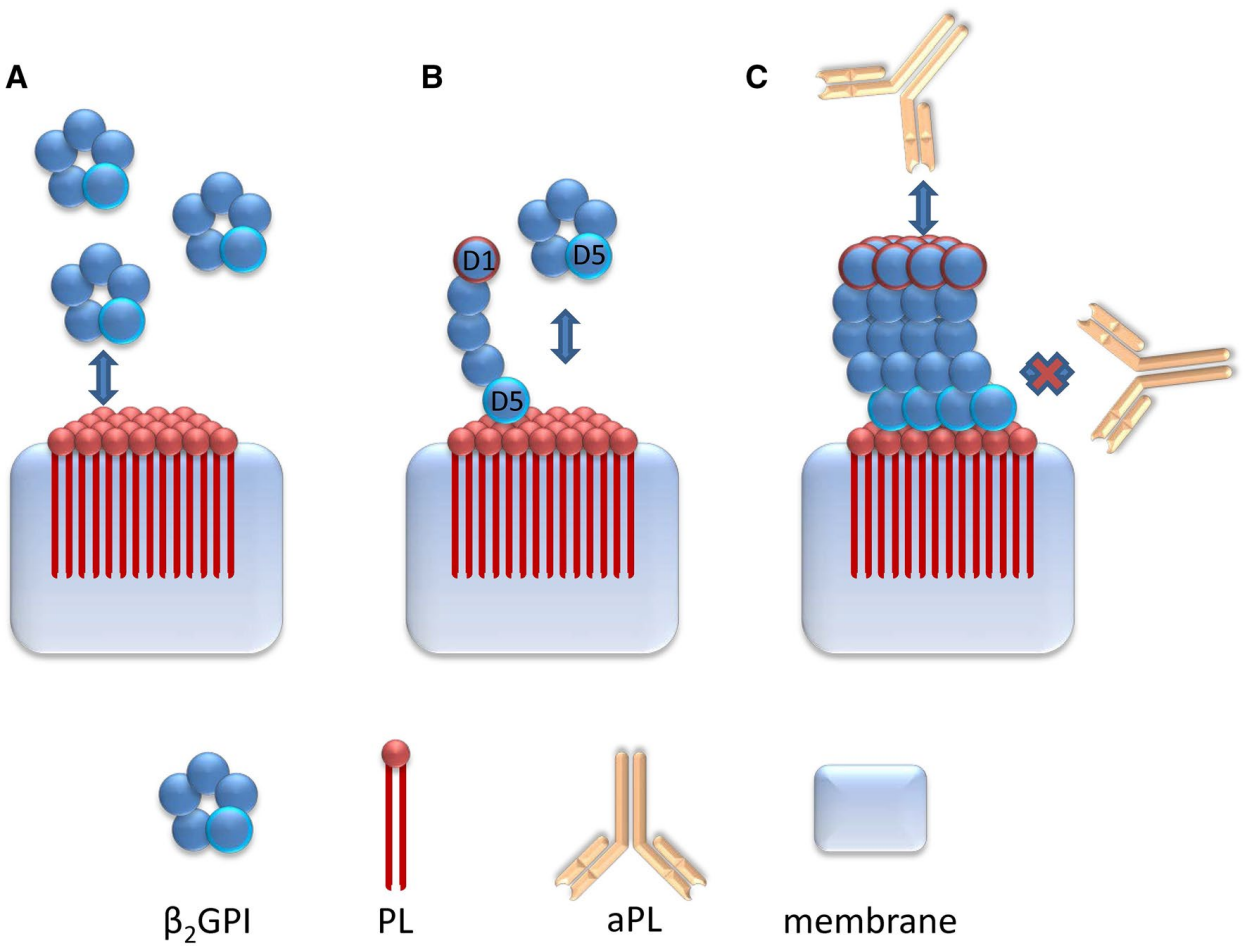
Preferential binding of anti-phospholipid antibodies (aPL) to domain 1 (D1) of patient’s beta2-glycoprotein I (β_2_GPI) in the line immunoassay (LIA). In contrast to the planar solid phase used in enzyme immunoassays, the porous hydrophobic LIA membrane incorporates the hydrophobic phospholipid (PL)-tail during immobilization. This shields the by far larger tail of the amphiphatic PL molecule from the reaction environment and, thus, prevents unspecific interactions. Number, orientation, and accessibility of anionic phosphate groups of the differing hydrophilic PL heads may influence the binding of the patient’s β_2_GPI (**a**) and consequently of the β_2_GPI-dependent aPL (**c**). After binding of β_2_GPI to the immobilized anionic PL by domain 5 (D5, containing the PL-binding site), D1 forms the accessible top of the induced fish-hook-like β_2_GPI structure (**b**). Due to the high density of negatively charged PL heads on the membrane, the formation of a β_2_GPI layer with a unique D1 epitope structure is assumed. The layer formation seems to hinder aPL binding to β_2_GPI epitopes close to D5 [16]

The novel LIA used a hydrophobic membrane for the immobilization of different phospholipids and co-factors [21, 22] (Fig. 1). In particular, negatively charged phospholipids could bind the patient’s own β2GPI which in turn interacted with APS-specific aD1. Thus, the specificity of this new multiplex reaction environment was reported to be superior to aPL consensus ELISA [23]. In a recent study comparing aPL testing by LIA with ELISA in APS patients, asymptomatic aPL-positive carriers, and infectious patients, the LIA demonstrated a better specificity, too [16].

The appearance of aPL in SARD patients without characteristic clinical signs of APS is poorly understood yet. Thus, we wondered whether aPL detected by LIA or consensus criteria ELISA could discriminate primary APS from SARD without clinical symptoms of APS.

## Methods

### Patients and control subjects

Thirty-four patients with primary APS including 14 with arterial and/or venous thrombosis, 16 females with obstetric APS suffering from pregnancy-related complications, and 4 having both clinical symptoms were diagnosed by characteristic international clinical and serological consensus criteria (Table 1). The patients were selected from a cohort routinely followed at the university hospital in Brescia. All patients demonstrated elevated levels of aβ_2_GPI IgG antibodies by an in-house ELISA. This inclusion criterion was chosen to study the specificity of these antibodies against different β_2_GPI domains.

**Table 1:** Demographic, clinical, and laboratory baseline characteristics of the 75 anti-beta_2_ glycoprotein I (aβ_2_GPI) IgG-positive patients and 20 healthy subjects (children)

	Primary thrombotic or obstetric APS (*n* = 34)	SARD (*n* = 41)	Healthy subjects (*n* = 20)
Sex, no. (%) female	31/34 (91%)	36/41 (88%)	9/20 (55%)
Autoimmune disease	34/34 (100%)	41/41 (100%)	0 (0%)
Primary APS	34/34 (100%)	0 (0%)	0 (0%)
SLE	0 (0%)	11/41 (27%)	0 (0%)
SSj	0 (0%)	2/41 (5%)	0 (0%)
SLE + SSj	0 (0%)	3/41 (7%)	0 (0%)
DLE	0 (0%)	1/41 (2%)	0 (0%)
PBC	0 (0%)	3/41 (7%)	0 (0%)
SSc	0 (0%)	2/41 (5%)	0 (0%)
DM/PM	0 (0%)	4/41 (10%)	0 (0%)
UCTD	0 (0%)	15/41 (37%)	0 (0%)
Thrombosis	19/34 (56%)	0 (0%)	0 (0%)
Arterial	7/19 (37%)	0 (0%)	0 (0%)
Venous	12/19 (63%)	0 (0%)	0 (0%)
Obstetric manifestations	20/34 (59%)	0 (0%)	NA
Pregnancy loss	15/20 (75%)	0 (0%)	NA
Preeclampsia	5/20 (25%)	0 (0%)	NA
Laboratory features			
LAC positivity	24/34 (71%)	18/41 (44%)	NP
aβ_2_GPI IgG, median OD (25–75th percentile)	1.470 (0.929–1.747)	1.004 (0.655–1.298)	0.139 (0.047–0.444)
aβ_2_GPI IgM, median OD (25–75th percentile)	0.350 (0.165–0.576)	0.450 (0.202–0.838)	0.088 (0.049–0.132)

*APS* anti-phospholipid syndrome, *aCL* anti-cardiolipin antibodies, *DLE* discoid lupus erythematosus, *DM*/*PM* dermato/polymyositis, *LAC* lupus anti-coagulant, *NA* not applicable, *NP* not performed, *OD* optical density, *PBC* primary biliary cirrhosis, *SARD* systemic autoimmune rheumatic disease, *SLE* systemic lupus erythematosus, *SSc* systemic scleroderma, *SjS* Sjögren syndrome, *UCTD* undifferentiated connective tissue disease

As disease controls, 41 patients with SARD and no anamnestic thrombotic and adverse pregnancy events but positivity for aβ_2_GPI IgG [11 with SLE, 2 with systemic sclerosis (SSc), 2 with Sjögren syndrome (SjS), 3 with SLE and secondary SjS, 15 with undifferentiated connective tissue disease (UTCD), 1 with discoid lupus erythematosus (DLE), 4 with dermato/polymyositis (DM/PM), and 3 patients with primary biliary cholangitis (PBC)] were enrolled. This group was chosen due to the comparability with the disease group. Furthermore, 20 healthy subjects (children) (HS) were included as non-diseased controls. All children were aPL negative. The study was approved by the local ethical committee after a written informed consent from each patient. All sera were stored at − 20 °C.

### ELISA for the detection of antibodies to cardiolipin and β_2_GPI

To detect classification criteria IgG and IgM antibodies to CL and β_2_GPI in the patient sera, commercially available solid-phase ELISAs employing purified human β_2_GPI in complex with CL and human β_2_GPI alone were used, respectively (GA Generic Assays GmbH, Dahlewitz, Germany). Assessment of aPL antibodies was conducted according to the instructions of the manufacturer [21]. The sera with a concentration equal or more than 10 U/mL for IgG and IgM, respectively, was considered positive. The same serum samples were also analyzed by in-house assays and the results were comparable with the commercial ELISA (data not shown).

Research ELISAs for aD1 and aD4–5 IgG developed by Inova Diagnostics (San Diego, US) were performed as previously described [9]. A ratio of aD1 and aD4–5 with a cutoff of 1.5 was used to test sera for aPL positivity.

### LAC testing

The analysis of lupus anti-coagulant (LAC) was performed according to the international recommendations [24]. Thus, the LAC testing comprised a three-step procedure:

- Demonstration of a prolonged phospholipid-dependent clotting time as screening test of hemostasis by dilute Russell viper venom time (dRVVT) or activated partial thromboplastin time (aPTT or lupus aPTT) analysis.
- Mixing patient plasma with normal plasma fails to correct the prolonged screening test(s).
- Addition of excess phospholipid shortens or corrects the prolonged coagulation test (demonstration of phospholipid dependence).

### Line immunoassay for the detection of aPL antibodies

Antibodies to CL, PA, PC, PE, PG, PI, PS, the protein co-factors β_2_GPI, AnV, and PT were detected using a commercially available LIA according to the recommendations of the manufacturer (GA Generic Assays GmbH) [16]. Processed LIA strips were read out densitometrically employing a scanner with the evaluation software Dr. Dot Line Analyzer (GA Generic Assays GmbH) and a grayscale calibration card for standardization. The grayscale calibration card was provided on the template of the kit. Values were read off as optical density (OD) units and OD values equaling or above 50 were scored positive. This cutoff was determined by calculating the 99th percentile of 150 apparently healthy individuals as recommended by the international classification criteria for aPL testing and Clinical and Laboratory Standards Institute (CLSI) guideline C28-A3 [25].

### Statistical analysis

Fisher’s exact test was performed with two-tailed probability to detect the differences between groups as appropriate using Medcalc statistical software (Medcalc, Mariakerke, Belgium). Inter-rater agreement statistics ware applied for within-group comparison. The two-tailed, Kruskal–Wallis test was used to test for statistically significant differences of independent samples. *p* values of less 0.05 were considered significant.

## Results

### Comparison of aPL analysis by ELISA and LIA

To identify the aPL antibody profiles by ELISA and LIA, we tested 34 sera from patients with APS and 61 controls including 41 asymptomatic patients suffering from SARD and 20 HS (Table2). Comparative analysis of the consensus criteria aPL aCL and aβ_2_GPI in 95 sera detected by LIA and ELISA demonstrated good agreement for IgG [kappa = 0.69, 95% confidence interval (CI) 0.55–0.84; 0.68, 95% CI 0.54–0.83, respectively] and moderate concordance for IgM (kappa = 0.52, 95% CI 0.35–0.69; 0.49, 95% CI 0.32–0.66, respectively). There was a significant difference according to McNemar’s test for aCL and aβ_2_GPI IgM (difference: 15.8 and 13.7%, *p* < 0.05, respectively), whereas the corresponding IgG analyses did not reveal a significant difference for both methods. The consensus ELISA testing for aCL and aβ_2_GPI is not significantly more specific than aPL analysis by LIA covering 10 aPL with regard to the false positives in HS (1/20 vs 3/20, *p* = 0.605).

**Table 2:** Anti-phospholipid antibody (aPL) positives by line immunoassay (LIA) and consensus criteria enzyme-linked immunosorbent assay (ELISA) in 95 subjects including 34 patients with anti-phospholipid syndrome (APS), 41 with systemic autoimmune rheumatic disease (SARD), and 20 healthy subjects (children) (HS) as control groups

ELISA						LAC	TP	aD1/aD4–5	LIA																				
	ACL		aβ_2_GPI		Any aPL				aCL		aPA		aPC		aPE		aPG		aPI		aPS		aAnV		aβ_2_GPI		aPT		Any aPL
	G	M	G	M					G	M	G	M	G	M	G	M	G	M	G	M	G	M	G	M	G	M	G	M	
APS *n* = 34	24	21	30	18	31	24	14	28	27	13	24	10	0	0	0	0	18	1	7	2	30	16	2	5	27	17	17	5	33
SARD *n* = 41	18***	19	25**	23	33	18***	8	26	26	12	22	8	0	0	0	0	4*	0	3	2	27***	9***	0	3	22***	12	7**	3	32
HS *n* = 20	NA	0^#^	0^#^	1^##^	1^#^	NA	NA	NA	1^#^	0^##^	0^#^	0^####^	0	0	0	1	0^#^	1	0^####^	0	0^#^	0^##^	0	0	1^#^	0^#^	1^##^	1	3^#^

Comparison of APS vs SARD: *p < 0.001, **p < 0.01, ***p < 0.05

Comparison of APS vs HC: ^#^*p* < 0.0001, ^##^*p* < 0.001, ^###^*p* < 0.01, ^####^*p* < 0.05

LAC and TP testing was conducted for 33 APS and 39 SARD patients only

*a*β_*2*_*GPI* anti-beta_2_ glycoprotein I, *aCL* anti-cardiolipin, *aD1* anti-domain 1, *aD4*–5 anti-domains 4 and 5, *aPA* anti-phosphatidic acid, *aPC* anti-phosphatidylcholine, *aPE* anti-phosphatidylethanolamine, *aPG* anti-phosphatidylglycerol, *aPI* anti-phosphatidylinositol, *aPS* anti-phosphatidyl-serine, *aAnV* anti-annexin V, *aPT* anti-prothrombin, *LAC* lupus anti-coagulant, *TP* triple positivity (aCL positive, *a*β_*2*_*GPI* positive, LAC positive), *NA* not available

### Comparison of aPL testing in APS patients and healthy controls

Both LIA and ELISA showed significantly higher prevalences of positive consensus criteria aPL (aCL and aβ_2_GPI IgG as well as IgM) in APS patients (*n* = 34) compared to HS (*n* = 20) (*p* < 0.05, respectively, Table 2). In addition, the LIA revealed significantly more prevalent aPA and aPS IgG as well as IgM, and further aPG IgG, aPI IgG, and aPT IgG in APS patients (*p* < 0.05, respectively).

### Comparison of qualitative aPL testing in APS patients and disease controls

The comparison of APS patients (*n* = 34) with asymptomatic SARD patients (*n* = 41) revealed significantly higher prevalences in APS patients for the criteria aPL aCL and aβ_2_GPI IgG detected by ELISA (*p* < 0.05, respectively, Table 2). The, frequency of LAC positivity was also significantly elevated in APS, whereas the frequency of triple positivity demonstrated a tendency only (*p* = 0.0312, 0.0713, respectively). In terms of aPL testing by LIA, aPG IgG, aβ_2_GPI IgG, and aPT IgG as well as aPS IgG and IgM were significantly higher prevalent in patients suffering from APS in contrast to SARD patients. Of note, aPG IgG showed a significantly lower prevalence of 9.8% in asymptomatic SARD patients (4/41) compared to 52.9% in APS patients (18/34, *p* < 0.0001). The same holds true for aPT IgG with a prevalence of 17.1% in SARD (7/41) vs 50.0% in APS (17/34, *p* = 0.0002).

### Comparison of quantitative aPL testing in APS patients and controls

**Fig. 2:**
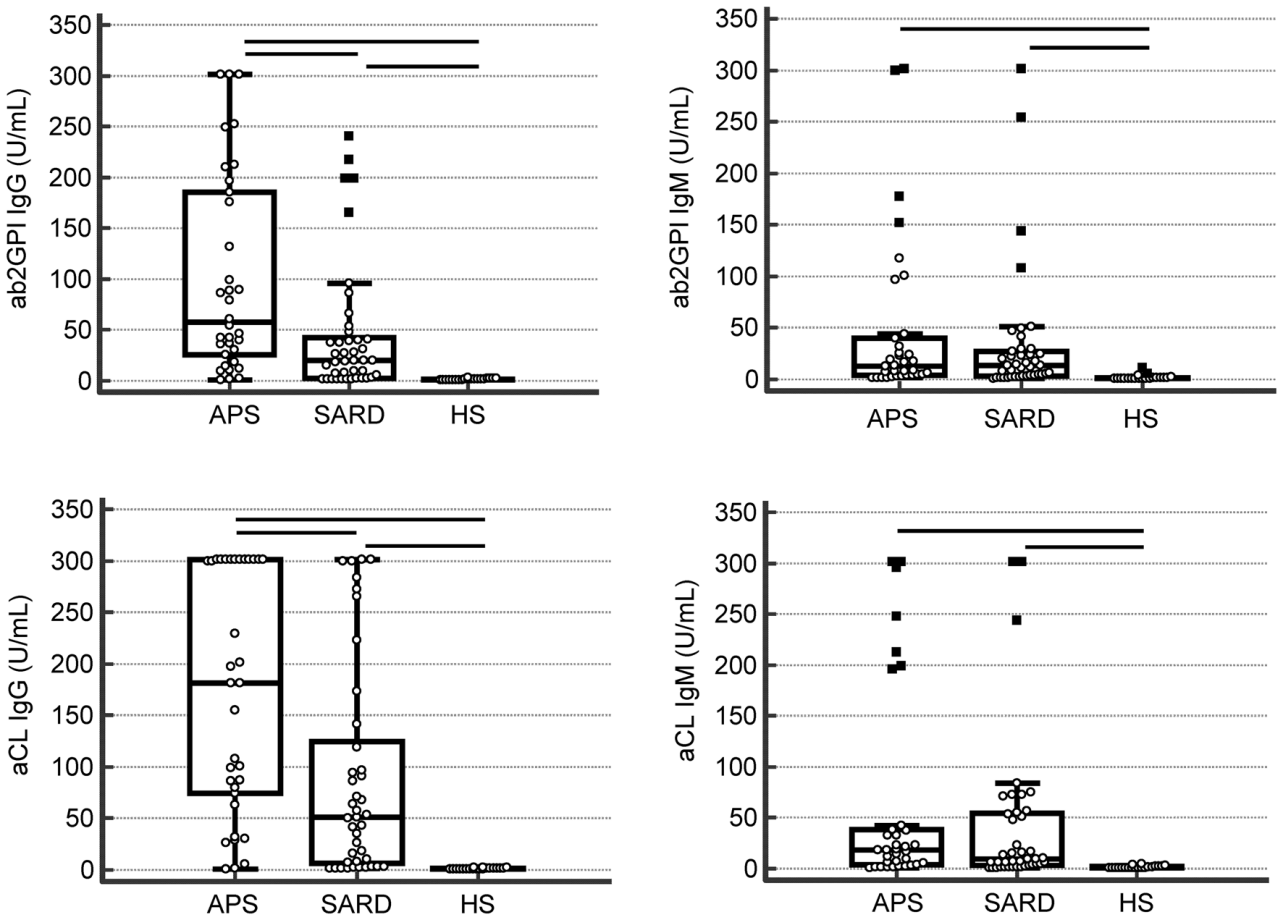
Consensus criteria anti-phospholipid antibodies detected by enzyme-linked immunosorbent assays (ELISAs) in 95 subjects including 34 patients with anti-phospholipid syndrome (APS), 41 with systemic autoimmune rheumatic disease (SARD) and no adverse APS events, as well as 20 healthy subjects (children) (HS) as control group. *aβ*_*2*_*GPI* anti-beta_2_-glycoprotein I, *aCL* anti-cardiolipin

Quantitative assessment revealed significantly different aPL IgG and IgM levels in the study cohorts regarding all consensus criteria aPL (aCL and aβ_2_GPI) by ELISA (Kruskal–Wallis, *p* < 0.05, respectively (Fig. 2). In terms of LIA testing, IgG and IgM to CL, β_2_GPI, PA, PS, PT, and PG as well as IgM to AnV demonstrated significantly different values (Kruskal–Wallis, *p* < 0.05, respectively).

**Fig. 3:**
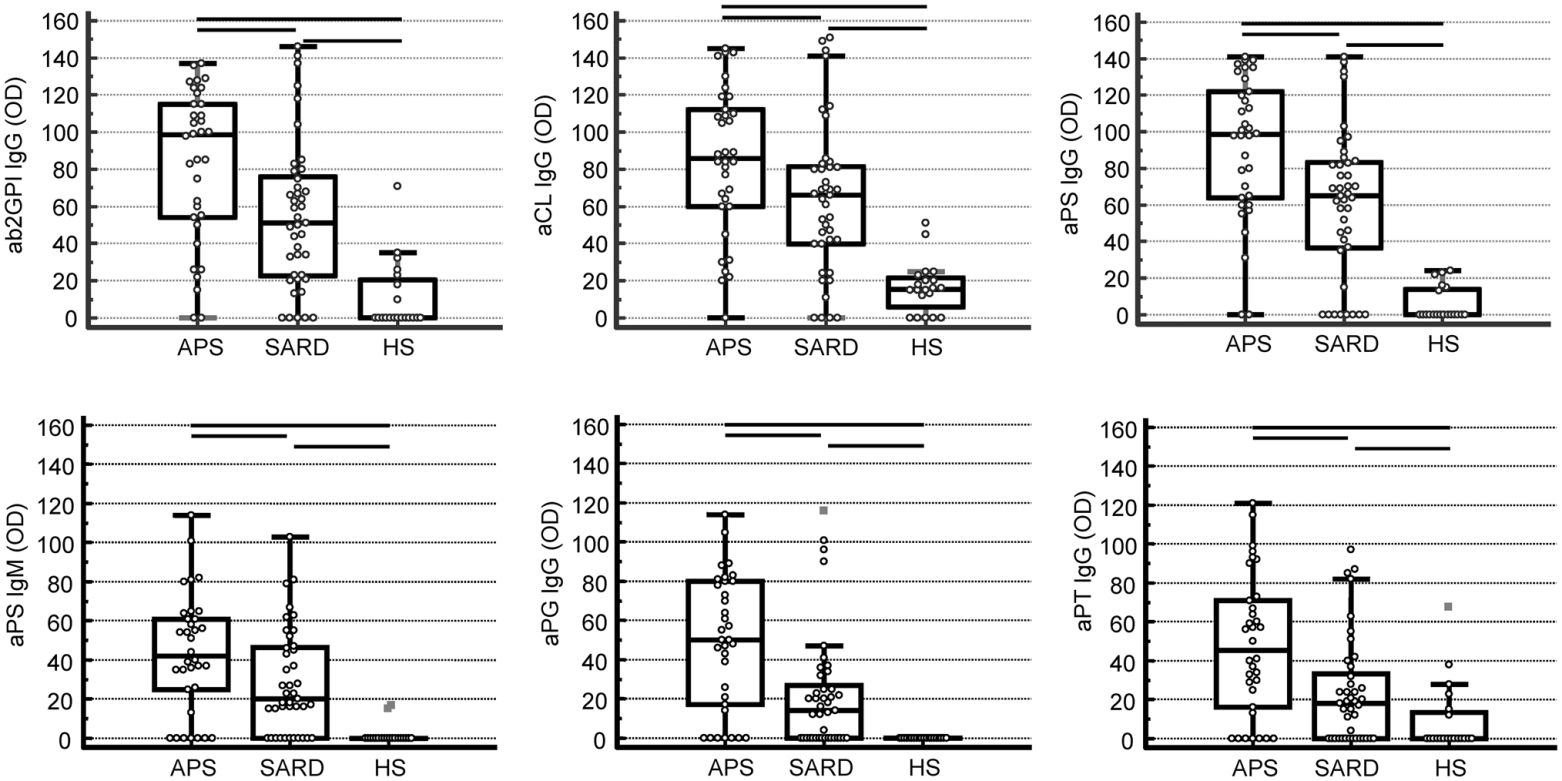
Anti-phospholipid IgG antibodies analyzed by line immunoassay (LIA) in 95 subjects including 34 patients with anti-phospholipid syndrome (APS), 41 systemic autoimmune rheumatic disease (SARD), and no adverse APS events, as well as 20 healthy subjects (children) (HS) as control group. *aβ*_*2*_*GPI* anti-beta_2_-glycoprotein I, *aCL* anti-cardiolipin, *aPG* anti-phosphatidylglycerol, *aPI* anti-phosphatidylinositol, *aPS* anti-phosphatidylserine, *aPT* anti-prothrombin, *OD* optical density

Regarding the differentiation of APS patients from asymptomatic SARD patients by consensus criteria ELISA, only aCL and aβ_2_GPI IgG revealed significantly different quantitative levels (post hoc analysis, *p* < 0.05, respectively) (Fig. 2). In addition to aCL and aβ2GPI IgG detected by LIA, IgG to PA, PS, PG, PT, and IgM to PS revealed significantly higher levels in APS patients, too (post hoc analysis, *p* < 0.05, respectively) (Fig. 3).

### Comparison of the assay performance of aPL detected by ELISA and LIA

**Fig. 4:**
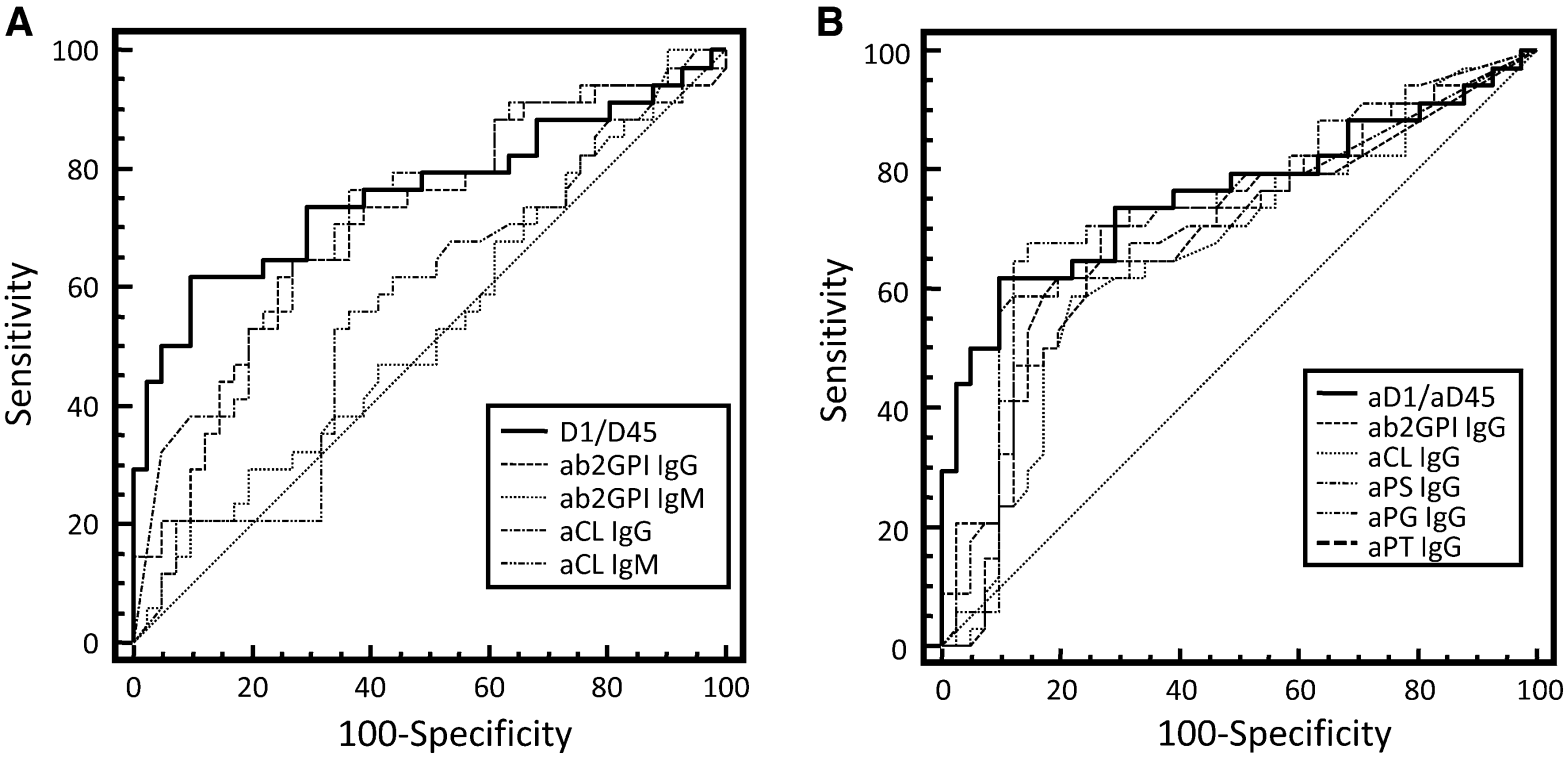
Receiver-operating characteristics curve analysis of anti-phospholipid antibodies (aPL) detected by enzyme-linked immunosorbent assay (ELISA) and line immunoassay (LIA) in 34 patients with anti-phospholipid syndrome and 41 disease controls without clinical APS symptoms. The ratio of anti-domain 1 (aD1) and D4–5 antibody reactivity (aD4–5) by ELISA was compared with criteria aPL determined by ELISA (**a**) and aPL IgG by LIA (**b**). *aβ*_*2*_*GPI* anti-beta_2_-glycoprotein I, *aCL* anti-cardiolipin, *aPG* anti-phosphatidylglycerol, *aPI* anti-phosphatidylinositol, *aPS* anti-phosphatidylserine, *aPT* anti-prothrombin

To compare the diagnostic performance for the differentiation of APS from SARD, receiver-operating characteristic (ROC) curve analysis was performed for aPL detected by ELISA and LIA (Fig. 4). The ratio of D1 to D4–5 reactivity demonstrated the best performance with an area under the curve (AUC) of 0.76 when compared with consensus criteria aPL ELISAs. The AUC of this ratio was significantly higher than the AUCs of aCL and aβ_2_GPI IgM (*p* < 0.05, respectively) (Table 3). However, there was no significant difference in the prevalence of the aD1/aD4–5 ratio in APS in contrast to SARD using the cutoff of 1.5 established in another study previously [9]. In accordance with the ROC curve analysis for the aD1/aD4–5 ratios of this study, a cutoff of 4.6 instead of 1.5 for the optimal differentiation of APS and SARD was required. Applying this new cutoff, there were 21/34 (61.8%) positive APS patients in contrast to 28/43 (82.4%) with the old cutoff of 1.5. Accordingly, the new cutoff lowered the prevalence of positives in the asymptomatic SARD cohort from 26/41 (63.4%) to 5/41 (12.2%). Consequently, the new prevalence of the APS cohort was significantly higher in contrast to the one of the SARD cohorts (*p* < 0.0001).

**Table 3:** Receiver-operating characteristics curve analysis of anti-phospholipid antibodies (aPL) detected by enzyme-linked immunosorbent assay (ELISA) and line immunoassay (LIA) in 34 patients with anti-phospholipid syndrome and 41 disease controls

aPL	AUC	SE	95% CI
ELISA			
aD1/aD4–5	0.760	0.0597	0.647–0.851
aβ_2_GPI IgG	0.705	0.0617	0.588–0.805
aβ_2_GPI IgM	0.534*,^$,§^	0.0679	0.416–0.651
aCL IgG	0.725	0.0598	0.609–0.821
aCL IgM	0.559*,^$,§^	0.0679	0.440–0.674
LIA			
aβ_2_GPI IgG	0.691	0.0638	0.574–0.793
aCL IgG	0.660	0.0651	0.541–0.765
aPS IgG	0.716^&^	0.0613	0.600–0.814
aPG IgG	0.723^&^	0.0630	0.608–0.821
aPT IgG	0.701	0.0628	0.584–0.801

Area under the curve (AUC) was determined for the ratio of anti-domain 1 of beta_2_ glycoprotein I (aD1) and domains 4–5 (D4–5) antibody reactivity (D4–5) by ELISA and compared with those for criteria aPL determined by ELISA and aPL IgG by LIA

*aβ*_*2*_*GPI* anti-beta_2_ glycoprotein I, *aCL* anti-cardiolipin, *aPG* anti-phosphatidyl-glycerol, *aPI* anti-phosphatidylinositol, *aPS* anti-phosphatidyl-serine, *aPT* anti-prothrombin, *CI* confidence interval, *SE* standard error

AUC comparison of ELISA

*p < 0.05 for the comparison to the AUC of the ratio of aD1 to D4–5

^$^*p* < 0.05 for the comparison to the AUC of aβ_2_GPI IgG

^§^*p* < 0.05 for the comparison to the AUC of aCL IgG

AUC comparison of LIA

^&^*p* < 0.05 for the comparison to the AUC of aCL IgG

Furthermore, there was no significant difference of the AUC for the aD1/aD4–5 ratio to the AUC of aPG IgG detected by LIA. The latter demonstrated in turn the best performance among the aPL IgG determined by LIA and was significantly higher than the AUC of aCL IgG (Table 3). In accordance with inter-rater agreement statistics, there was a good agreement for aPG IgG with the aD1/aD4–5 ratio [kappa = 0.71, 95% confidence interval (CI) 0.52–0.89] and no significant difference (McNemar’s test: difference = 5.3%, 95% CI − 6.8 to 15.7%, *p* = 0.4807).

In contrast, the strength of agreement of the aD1/aD4–5 ratio with all four consensus criteria aPL determined by ELISA was only fair (kappa < 0.4, aCL IgM, and aβ_2_GPI IgG) or poor (kappa < 0.2, aβ2GPI IgM, and aCL IgG).

## Discussion

The persistent occurrence of aPL was the serological hallmark of APS and was defined as a mandatory classification criterion [26]. It is a well-accepted consensus that APS-specific aPL interact with phospholipid-binding proteins such as β_2_GPI or complexes thereof with phospholipids. Among aPL, the correlation of aβ_2_GPI with clinical symptoms appeared to be the strongest one [2, 20, 27]. In this context, aPL binding to D1 and not to D4–5 of β_2_GPI has been the basis for the detection of disease-specific aPL [9, 28]. There has been no single assay to assess all different aPL subpopulations, and thus, aCL, aβ_2_GPI, and LAC testing have been recommended to identify all the potential aPL. Triple positivity has been considered a risk factor and could be used for stratification of APS patients [29].

A novel aPL assay technology employing a hydrophobic membrane for aPL profiling by LIA was reported recently [19]. The LIA membrane provided a unique matrix allowing phospholipids to mimic their natural conformation required for co-factor binding as reported for other amphiphatic non-protein antigenic molecules [30–32]. In particular, D1 of patient’s serum β_2_GPI appeared to be presented in the LIA reaction environment for APS-specific aPL binding more favorably than the corresponding D4–5 after the interaction of serum β_2_GPI with the immobilized phospholipids (Fig. 1). Interestingly, complexes of the patient’s β_2_GPI with differing immobilized phospholipids demonstrated different aD1 reactivity. Altogether, this seemed to support the differentiation of disease-specific aPL in APS patients from aPL found in individuals with infectious disease or in asymptomatic carriers [16]. Thus, we attempted to ascertain whether this novel reaction environment for the multiplex detection of aPL can discriminate aPL in APS patients from those occurring in patients with other autoimmune disorders like SARD not demonstrating clinical symptoms of APS.

The agreement of aPL testing by LIA with consensus criteria aPL by ELISA was good (IgG aPL) to moderate (IgM aPL) and, thus, was in line with previously published comparative data [16, 21]. In addition, the favorable specificity of aPL testing by LIA could also be confirmed in this study revealing no significant difference for all ten aPL tested by LIA compared with the four consensus criteria aPL by ELISA.

In terms of the occurrence of aPL in APS patients compared with that in SARD patients without clinical symptoms of APS, only IgG consensus criteria aPL demonstrated a significant difference. Although LAC testing revealed significantly different prevalences too, triple positivity analysis did not differentiate APS patients from those with SARD. Of interest, aPL IgG by LIA also revealed significantly higher prevalences in APS compared to SARD without clinical symptoms of APS. In contrast to aCL IgG by ELISA, however, the difference of aCL IgG by LIA did not reach significance. Of note, aPS testing demonstrated for both immunoglobulin isotypes significantly different prevalences. Provided that the positive aPS result was due to interaction of patient’s aPS with serum β_2_GPI of the patient sample having bound to immobilized PS on the LIA membrane, this might add further evidence to the assumption that β_2_GPI binding to negatively charged phospholipids induces specific conformational changes unique for each distinct phospholipid. Since D1 binding by aβ_2_GPI was preferred in the LIA reaction environment [16], the differing accessibility of respective epitopes on D1 could determine the specificity of such aPL reactivity. Thus, the significantly reduced prevalence of aPG IgG in SARD without clinical symptoms of APS (9.8%) compared to APS (52.9%) in this study is of particular interest in this context. Of note, CL also referred to as diphosphatidylglycerol represents a dimer of PG and CL’s head bears two phosphate groups forming a dianion for β_2_GPI binding [33]. This particular setting could induce a β_2_GPI configuration enabling sensitive binding of aPL but, obviously, did not provide a reaction environment for the discrimination of aPL occurring in APS and asymptomatic SARD patients. However, quantitative aPL testing did reveal significantly different levels of aCL IgG by LIA like did quantitative aPA, aPS, aPG, and aPT IgG analysis by this method. Of note, quantitative aPS IgM testing did corroborate the significant difference of the qualitative one. In contrast, consensus criteria’ aPL IgM analysis by ELISA did not reveal significantly different aPL levels in APS and asymptomatic SARD patients. This further highlights the specificity of the LIA reaction environment for aPL analysis and the putative role of differing β_2_GPI configurations for specific aPL binding.

Recently, the ratio of aD1 to aD4–5 was reported as a useful marker for APS [9]. Surprisingly, the recommended cutoff of aD1/D4–5 did not enable differentiating APS from SARD patients in this study. Only after applying ROC curve analysis and readjusting the cut-off to 4.6, significantly different prevalences in both groups were determined. Furthermore, the ROC curve analysis of the aD1/aD4–5 ratio revealed the best performance compared with the consensus criteria aPL determined by ELISA.

The assay performance of aPG IgG analysis being the best amongst the aPL detection by LIA was not significantly different from the performance of the aD1/aD4–5 ratio. In addition, there was no significant difference and a good agreement between qualitative aPG IgG analysis by LIA and D1/D4–5 ratio assessment. Thus, the LIA reaction environment consisting of immobilized PG interacting with the specimen’s β_2_GPI might favor the specific binding of aD1 as shown for aPL IgG testing recently (Fig. 1) [16]. Furthermore, a specific conformation of the bound β_2_GPI as a single molecule or of a β2GPI layer could minimize the binding of aD4–5 and, thus, bring about the high specificity of aPG analysis. As a fact, aD4–5 did not seem to be associated with the APS phenotype [7]. A similar LIA reaction environment with CL (diphosphatidylglycerol) instead of PG demonstrated a significantly poorer assay performance which underlines the importance of the phospholipid nature for specific aPL binding.

Our study has certain limitations. The relatively low number of samples results in large confidence interval (CI) in particular regarding the comparison of AUC values by ROC analysis. Furthermore, there could be a selection bias for patients with SARD, since these patients were recruited by aβ_2_GPI IgG positivity. We employed research ELISA for aD1 analysis which might differ from the presently available commercial aD1 assays.

In summary, aPG IgG analysis by LIA and assessment of the aD1/aD4–5 ratio by ELISA enabled the discrimination of aPL in patients with APS from those with asymptomatic SARD. This finding could be helpful in clinical practice, mainly because patients at risk of adverse APS events could be identified and managed more appropriately by primary prophylaxis and more frequent clinical controls [34].
